# Designing a Medical Tourism Website: A Qualitative Study

**Published:** 2017-02

**Authors:** Mahnaz SAMADBEIK, Heshmatollah ASADI, Mohammad MOHSENI, Afsaneh TAKBIRI, Ahmad MOOSAVI, Ali GARAVAND

**Affiliations:** 1. Dept. of Health Information Technology, School of Allied Medicine, Lorestan University of Medical Sciences, Khorramabad, Iran; 2. Dept. of Health Management and Economics, School of Public Health, Tehran University of Medical Sciences, Tehran, Iran; 3. Health Management and Economics Research Center, Iran University of Medical Sciences, Tehran, Iran; 4. *Dept. of* Health and Community Medicine, Dezful University of Medical Sciences, Dezful, Iran; 5. Dept. of Health Information Management, School of Management and Medical Informatics, Shiraz University of Medical Sciences, Shiraz, Iran

**Keywords:** Medical tourism, Information needs, Website, Hospitals

## Abstract

**Background:**

Informing plays a prominent role in attracting medical tourists. The enjoyment of proper medical information systems is one of the most important tools for the attraction of medical tourists. Iran’s ability in designing and implementing information networks has remained largely unknown. The current study aimed to explore information needs for designing a medical tourism website.

**Methods:**

This qualitative study was conducted in 2015 for designing Hospital Medical-Tourism Website (HMTW). A purposive sampling method was used and data were gathered using a semi-structured questionnaire. Totally, 12 faculty members and experts in the field of medical tourism were interviewed. Data were analyzed using the MAXQDA10 software.

**Results:**

Totally 41 sub-themes and 10 themes were identified. The themes included the introduction of hospital, general guide for patients, tourism information, information related to physicians in hospital, costs, treatment follow-up, online hospital appointment scheduling in website, statistics and news of hospital medical tourism, photo gallery and contacts. Among the themes, the participants highly emphasized four themes including costs (100%), tourism information (91.6%), information related to physicians in hospital, (83.3%) and treatment follow-up (83.3%).

**Conclusion:**

This profitable industry can be developed through considering information requirements for hospital medical tourism website.

## Introduction

Nowadays, tourism industry is one of the most important economic activities. In recent decades, we have witnessed the increasing growth of the global tourism market (1). Such a global expansion of tourism in industrialized and developed countries has created economic profits and high employment in many sectors related to production, from construction to agriculture and telecommunications (2). Based on the prediction by the World Tourism Organizations (WTO), approximate values of tourist travels will reach US$ 2 trillion per year by 2020 (3). Medical tourism is also a subset of the tourism industry, thus medical tourism is one of the areas of the modern tourism considered as one of the most profitable and competitive industries in the world. Many governments wish to benefit from the economic advantages resulting from this industry and a growing competition has started between different countries, especially Asian developing countries, to attract medical tourists (4).

Medical tourism is an organized trip from the individual’s living environment to another place to maintain, improve and recover the individual’s physical and mental health (5). This type of tourism is associated with direct intervention of medical issues, and it is expected that, the results of such trips to be fundamental and long-term. Medical tourism addressed needs of people who are increasing in number day by day; these people can be tourists and patients (6). The past few years, the number of people exited from their country to utilize health services has increased (7).

Medical tourism included a wide range of medical care from oral care to cosmetic surgical procedures (8). All consumers of medical tourism services seek these services in order to benefit from the most up-to-date health care services along with proper aftercare treatment as well as to avoid waiting lists for treatment (9).

Iran, given its advantages regarding medical tourism, such as low cost, high quality of health services, competent physicians and abundant natural attractions, has decided to take these advantages (10). Therefore, considering the state of medical tourism, doing investment, identifying, resolving challenges and obstacles attracting tourist are inevitable. One of the most important tools for attracting medical tourists is to have appropriate medical information systems (11). One of the major obstacles in the development of medical tourism is the existence of invalid statistics and information in financial and medical fields (12, 13).

The lack of recording and statistics system mentioned as well as the lack of information and its retrieval as the main obstacles to the development of the dentistry tourism (14). Some provided suggestions to improve the compliance level of hospitals for developing medical tourism industry in hospitals in Isfahan. These include registering contents of medical records and paraclinical reports in English and according to international standards, designing and establishing hospital’s website in accordance with international standards, providing international telephone services and access to the World Wide Web. They also recommended that electronic and non-electronic service package should be prepared along with services’ tariff in patients’ language (15). Human resource considered development as well as information and marketing systems development as requirements to promote the medical tourism industry (16). The indispensable role of medical information and the existence of websites related to medical tourism in developing the medical tourism have been mentioned in all the studies conducted by researchers. Given the significant advances of medical sciences in various fields in Iran, abundant tourism attractions, high political and social security in Iran and low costs of medical services, compared to other countries, Iran should become one of hubs of the tourism industry in the world.

The authorities have not taken adequate actions to introduce the power of medical tourism and to design and utilize information networks (17), and Iran’s ability in this field has been largely unrecognized, while informing plays a significant role in attracting medical tourists (18). Thus, the current study aimed to determine information for designing hospital medical tourism website.

## Methods

This study was conducted to provide a minimum set of data required for designing hospital medical tourism website, in 2015 with using the purposive sampling. Samples were selected based on research goals, (19) purposefully, and specific information needed. Therefore, the issue people selected later is affected by who previously interviewed information they provided (20). Data saturation was reached by nine interviews, but to ensure further confidence up to 12 interviews were enrolled. Participants included four activists in Shiraz medical tourism, who initiate to attract tourist in private hospitals (one anesthesiologist, one pathologist, one hospital manager and one BSc nurse), six faculty members of the School of Health Management and Information Sciences, and 2 IT specialist (with PhD and master’s degree in management of information systems). Semi-structured interviews and interview guide were used to collect data. An interview guide on medical tourism websites available in developed countries was presented to the participants. That is an essential tool to help a researcher to extract facts, attitudes, processes, and opinion of subjects (20). The interview questions were semi-open questions. In semi-structured interviews and semi-open questions, the researcher and participants are free to talk further about the issue or question. This method allows researchers to assess the issue in more detail and ask participants more questions in addition to the questions included in the interview guide. In addition, researchers can discuss issues that arise regarding the issue during interviews (21). Interviews lasted 40–50 min.

Content analysis method is a proper method for obtaining reliable and valid data from text data (22). That involves five steps: orientation, knowledge of the conceptual framework, coding, charting and mapping interpretations (23).

Furthermore, the conventional approach was utilized in this study as a content analysis approach (24). First, the researchers to reach a general perception of the interviews once read the transcribed interviews; then again were read verbatim to identify codes. The final analysis was carried out and the relation between various codes and themes were determined.

Data analysis was performed using the MAXQDA10 software. To ensure the principle of data immersion, the interviews were listened several times and then were transcribed. To ensure the accuracy in the interpretation of the findings and data credibility, enough time was allocated to each interview; agreement on codes and checking the transcribed interviews were done by the researchers (Peer check), and participants verification (Member check) was also used.

Interviews were conducted in a quiet environment and in terms of the time, interviewees agreed it. Interviews were recorded after obtaining respondent’s consent. To ensure that the researchers had same perceptions of participants’ statements, the transcribed interviews were given to them for verification. In addition, the individuals participating in the study were assured that all information would remain confidential until the end of the study. When analyzing the data, instead of mentioning the personal characteristics of participants, a code was given to each questionnaire. Finally, data were categorized and analyzed and information for the medical tourism website was identified.

## Results

There are shortages and problems in the field of medical tourism in Iran, including:

- Low ability in the field of the application of information and communication technology;
- Low ability regarding the status of facilities;
- Weakness in coordination and communication between organizations related to health tourism (such as: travel agencies, hotels, insurance companies, cultural and historical sites);
- The lack of scientific and targeted planning for attracting medical tourists;
- The weakness in the use of modern advertising;
- The lack of staff fluent in international language in the majority of hospitals;
- Low medical standards in some hospitals;
- The lack of attention to and weakness in the introduction of the country’s abilities in order to attract medical tourists (1, 16, 25, 26).

The interviews about HMTW should include 10 main themes and several sub-themes, HMTW information needs are provided in Table 1, and then we have explained some of the most important ones.

**Table 1:** Information needs of HMTW

**Items**	**Capabilities of items**	
Introducing the Hospital	Presenting the history of the hospital from the beginning till now	
	Direction of hospital floors	
	Introducing hospital officials (dean, director, etc.)	
	Modern equipment available in hospital	
	Introducing the hospital sections and specialties available in the hospital	
	Presenting hospital vision and mission	
General guide for patients	Patients’ Bill of Rights	
	Providing basic medical information to patients	
	The introduction of useful medical links	
	Compliance with principles of hygiene in hospitals	
Tourism information^*^	Introduction of tourism attractions in the city and province	
	Providing maps for accessing to hospital and tourism sites	
	Introducing hoteling services in hospital for foreign patients	
	Introduction of hotels, restaurants and other well-known entertainment places in the city	
Information related to physicians in hospital^*^	The introduction of specialists and subspecialists in hospitals	Physician license verification
		Type of specialty and place of graduation
		Received academic honors and prestigious awards
	Ability to search for names and specialties	
	The attendance schedule of physicians in clinics of hospital	
	Address and contact number of hospital physicians’ offices	
	Presenting costs of global surgeries (pre-determined)	
Costs^*^	Comparing the hospital prime costs with other competing countries	
	Formulas to calculate respective costs	
	List of insurance contracts for tourists	
	Non-in person follow-ups after treatment	
Treatment Follow-up^*^	Tracking Complaints	
	Coordination ways to readmission	
	Medical consultations	
	Registration of requests and specialized questions	
Online hospital appointment scheduling in website	The possibility for registering people	Various clinics
		Para clinic sectors
	Providing tracking code	
	Possibility to change or cancel appointment time	
Statistics and News of HMTW	Statistics of foreign clients	
	Continuous monitoring of foreign patients’ satisfaction and presenting it in the related link	
	The newest news related to surgeries and other medical procedures	
	The schedule of journal clubs and scientific meetings in hospital	
Photo Gallery	Photos of the sectors in hospital	Operating rooms
		Overview of hospital
		Photos of surgeries
	Photos of tourism attractions	
	Photos of facilities as well as clinical and paraclinical services	
Contacts	Hospital contacts numbers	
	Hospital Email Address	
	Hospital exact address	
	Hospital Fax number	

### Costs:

All respondents believed that cost is a key part of the information and can be effective in attracting health tourists.

In this regard, one of the participants, “By evidence and documentation, we must prove to individuals and users that our hospitals are cheaper and the quality of services is higher than other hospitals in the region and competing countries in the region, so preparing a list of hospital costs compared with the prices of hospitals in other countries is needed, of course, this information should be continuously updated” (Participant No. 11).

### Tourist information:

Most of the participants (91.6%), believed that the tourist information is one of information needs for designing HMTW. For example, “Appropriate and worthy introduction of tourism attractions is as important as having them to benefit from these attractions, and because these sites are available to everyone and these attractions should be presented well (Participant 3).

### Information related to physicians in hospital:

“For example, there may be a prominent physician and many people choose the hospital due to the physician, because one of the main reasons for referring to a hospital is a specific doctor in that hospital. Moreover, having physicians’ information can be useful for patients in terms of the degree of specialization of that physician (specialist or subspecialist)” (Participant 1). In this regard, 83.3% of participants agreed and regarded this information to be important.

### Treatment follow-up:

“Patients should ensure that they will not be left after treatment and hospital take their responsibility. In addition, patients’ treatment follow-up removes the need the physical presence of patients and their families in the hospital where they received services, and patients by referring to the website, can find out what to do after the treatment, for example, how to sit and walk after some treatment procedures may be important” (Participant 5). 83.3% of the respondents believed that treatment follow-up should be considered in the design of medical tourism website.

Introducing the hospital, general guide for patients, statistics and news of hospital medical tourism, photo gallery, contacts and online hospital appointment scheduling in website were other factors recommended by participants to be considered in designing medical tourism website. In addition, most of the participants in the study evaluated costs item as the most important item on the website while they evaluated the online appointment-scheduling item as the least important item for foreign medical tourists.

The most important general characteristics considered when designing a website for medical tourism are the ability to translate contents in different languages including the language of target countries (Arabic, Turkish, English, Kurdish, and Pashto), proper graphics, structural stability, loading speed, and updating website content. Overall, the purpose of the participants is to respect the principle of user-friendly in designing and using the website.

## Discussion

Many studies about medical tourism in the world have emphasized the importance of medical information systems in the development and growth of this industry (27–30). Therefore, this tool should be used to advance macroeconomic goals and objectives. However, to have a strong tourism industry, we need the information systems to introduce it to audiences, and most studies have focused on the positive impact on the development of medical tourism (11–16). Another important point is that, Iran has no place between the top countries with accredited hospitals (JCI) (Table 2).

**Table 2:** Top ten hospitals with JCI standard (31)

**Country name**	**The number of accredited hospitals with JCI**
Turkey	42
Saudi Arabia	39
United Arabic Emirates	38
Brazil	21
Ireland	17
Thailand	17
India	16
Italy	15
Singapore	14
China	14

The countries in the region, including UAE, Saudi Arabia, and Turkey have a more advanced position than Iran. From the results of previous studies (12, 13), information systems have high power in developing medical tourism industry and by using them, this development can be achieved and competition at the international and regional levels can be realized.

Websites are the most important means of medical information. The existence of HMTW will greatly help the development of medical tourism (12, 13). Most people in Canada get the information about medical tourism on the Internet and websites, and medical companies are deal with international marketing through designing related websites (32). The Ministry of Health has passed a six-article act in order to improve the tourism industry, which its sixth article is related to conditions and criteria for medical centers website (25). However, designing and using HMTW, in turn, require the collection of a set of certain information. Low costs are the main incentives for tourists (26). As presented in Table 3, many Asian countries provide a list of important operations and surgeries and compare their costs with other competing countries.

**Table 3:** Comparison of surgeries cost (US $) in some leading countries in health tourism (4)

**Procedure**	US Retail Price^*^	US Insurers’ Cost^*^	India^*^	Thailand^*^	Singapore^**^	Iran^***^
Heart bypass	210,842	94,277	10,000	12,000	20,000	37,614
Heart valve replacement	274,395	122,969	9,500	10,500	13,000	46,228
Angioplasty	98,618	44,268	11,000	13,000	13,000	16,105
Hip replacement	75,399	31,485	9,000	12,000	12,000	31,184
Knee replacement	69,991	30,358	8,500	10,000	13,000	30,982
Spinal fusion	108,127	43,576	5,500	7000	9,000	-

* Retail price and insurers’ costs represent the mid-point between low and high ranges.

** U.S. rates include at least one day of hospitalization; international rates include airfare, hospital and hotel.

*** Information about Iran added to the above table by researchers of the current study. The costs calculated based on the average amount of nine medical records and the prices related to surgeries and hospitalization costs while costs of hotel and airfares were not included.

According to Table 3, audience is attracted to Indian health centers because medical costs are much lower than other countries. In addition, factors affecting the choice of other countries for the treatment and travel, authors concluded that costs also affect the choice of tourist destination (33). Prices related to surgeries are the most important dimensions for attracting tourists via websites for tourist attraction (34). The issue of costs and informing about costs are separate issues. Thus, Iranian hospitals can provide websites with such tables to attract medical tourists. The part related to the treatment follow-up is one of the important sections related to HMTW. To improve medical tourist attraction, Jordan has developed plans for follow-up after treatment (25). Besides, 52.9% of websites of tourist attraction in Canada included the option related to treatment follow-up in the websites (34). In many cases, the procedure of treatment of patients in hospitals or other medical centers may be long and lead to readmission of people. In such circumstances, authorities should adopt measures to communicate with patients and the hospital. By including this section in the website for medical tourism, the hospital can facilitate the reconnection of individuals to the hospital, and provides the possibility for teleconsultation and other cases such as taking medicines. The possibility of readmission and the ease of this process will be effective in patients’ psychological security (35).

The part related to information on physicians in hospital, is other important sectors in the medical tourism website that may have significant impact on the patients’ decision to choose a hospital as their destination. The access to information such as physicians’ degree has effect on patients’ decision regarding selecting hospital (36). Given the existence of proficient and competent physicians at global and regional levels in Iran (10), the context for the development of medical tourism is available, however, informing this should be done well; and information about medical team, particularly physicians as leaders of the team should be informed accurately on the website.

One of the most important sectors of medical tourism website is tourism information that by proper presenting it, many tourists can be attracted. In Canada, medical tourism companies on their websites and social networks such twitter, youtube, and Facebook have attempted to attract tourists by introducing tourism attractions and medical services at the international level (32). Results of another study are consistent with those of the current one; providing information related to tourism opportunities is one of key pillars in decision making on selecting the destination hospital by patients (37).

In designing and using medical tourism website, hospital appointment scheduling arrangements should be considered for visitors. Centers providing services use online appointment scheduling to manage the access to service provision, because the online appointment scheduling system reduces waiting time effectively and therefore, increases patient satisfaction with the center providing services (38). Long waiting time for receiving medical services was significant factors for patients (39).

On the HMTW, methods of contact such as telephone fax and e-mail should be provided to clients, because in the medical tourism industry communication must be available 24 h a day, 7 d a week (40). Therefore, besides website, a translator and a 24-h telephone line should be established in a hospital. Experts participating in the study emphasized presenting photos of tourism sites and hospital facilities on the HMTW. Hospitals active in the area of medical tourism mainly display pictures of hospitals’ overview, diagnostic images and photos of the hospital equipment on their website, and publish them on the internet and other media to attract more medical tourists (41).

Items including the introduction of hospital, statistics as well as general guide for patients should be considered in designing the HMTW. In designing HMTW, issues such as the ability to convert to various languages such as languages of target countries, proper graphics, structural stability, loading speed, updating and the content of the website should be considered. Given that, Universities of Medical Sciences in the country have not had successful performance in designing, maintaining and using websites (42), therefore, accurate and complete studies on designing and using websites in the health system and particularly HMTW should be conducted.

## Conclusion

Designing proper medical tourism websites for hospitals is a good opportunity to attract health tourists for the country. Websites in a virtual space act as a gateway that people can easily select their destination with high quality, low price and proper services. Therefore, authorities make the necessary decisions to apply hospital medical tourism websites.

## Ethical considerations

Ethical issues (Including plagiarism, informed consent, misconduct, data fabrication and/or falsification, double publication and/or submission, redundancy, etc.) have been completely observed by the authors.

## Appendix 1: The organizational position of interviewees

**Table:**

**Type of activity**	**Field of study**	**Organizational position**
Activist in medical tourism	Anesthesiologist	President of hospital
Activist in medical tourism	PhD in Anatomy	chief executive officer of hospital
Activist in medical tourism	Clinical pathology specialist	Chairman of the hospital board
Activist in medical tourism	BSc in nursing	Activity in the field of attracting patients from other countries
Faculty members of the School of Health Management and Information Sciences	PhD in Healthcare services management	Faculty member
Faculty members of the School of Health Management and Information Sciences	PhD in healthcare services management	Faculty member and chief executive officer of hospital
Faculty members of the School of Health Management and Information Sciences	PhD in health information management	Faculty member
Faculty members of the School of Health Management and Information Sciences	PhD in health information management	Faculty member
Faculty members of the School of Health Management and Information Sciences	PhD in health information management	Faculty member
Faculty members of the School of Health Management and Information Sciences	PhD in health information management	Faculty member
Health information technology expert	PhD in information systems	Faculty member
Health information technology expert	MSc in information technology management	Hospital employee

## Appendix 2: Interview Questions

The main questions included three questions as below; also the participants were asked to answer the questions raised according to the flow of the interview.

In your opinion, what items should be included on a website for hospitals active in medical tourism field? Please explain them. Among items you have mentioned, what items are more important? Please explain them.

In your opinion, what are general features, which should be taken into account when designing a medical tourism website?
